# Cognitive Performance and Exposure to Organophosphate Flame Retardants in Children: Evidence from a Cross-Sectional Analysis of Two European Mother–Child Cohorts

**DOI:** 10.3390/toxics11110878

**Published:** 2023-10-24

**Authors:** Valentina Rosolen, Elisa Giordani, Marika Mariuz, Maria Parpinel, Vicente Mustieles, Liese Gilles, Eva Govarts, Laura Rodriguez Martin, Kirsten Baken, Greet Schoeters, Ovnair Sepai, Eva Sovcikova, Lucia Fabelova, Jiři Kohoutek, Tina Kold Jensen, Adrian Covaci, Maarten Roggeman, Lisa Melymuk, Jana Klánová, Argelia Castano, Marta Esteban López, Fabio Barbone

**Affiliations:** 1 Central Directorate for Health, Social Policies and Disability, Friuli Venezia Giulia Region, Via Cassa Di Risparmio 10, 34121 Trieste, Italy; 2 Department of Medicine, University of Udine, Via Colugna 50, 33100 Udine, Italy; 3 Center for Biomedical Research, University of Granada, 18012 Granada, Spain; 4 Instituto de Investigación Biosanitaria de Granada, 18012 Granada, Spain; 5 Consortium for Biomedical Research in Epidemiology and Public Health, 28029 Madrid, Spain; 6 VITO Health, Flemish Institute for Technological Research (VITO), 2400 Mol, Belgium; 7 BrabantAdvies, Brabantlaan 3, 5216 TV ‘s-Hertogenbosch, The Netherlands; 8 Department of Biomedical Sciences & Toxicological Centre, University of Antwerp—Campus Drie Eiken, Universiteitsplein 1, Wilrijk, 2610 Antwerp, Belgium; 9 Toxicology Department, Science Group, UK Health Security Agency, Harwell Science and Innovation Campus, Didcot OX11 0RQ, UK; 10 Department of Environmental Medicine, Faculty of Public Health, Slovak Medical University, 83303 Bratislava, Slovakia; 11 RECETOX, Faculty of Science, Masaryk University, Kotlářská 2, 611 37 Brno, Czech Republic; 12 Department of Clinical Pharmacology, Pharmacy and Environmental Medicine, Institute of Public Health, University of Southern Denmark, 5000 Odense, Denmark; 13 Toxicological Centre, University of Antwerp, Wilrijk, 2610 Antwerp, Belgium; 14 National Centre for Environmental Health, Instituto de Salud Carlos III, 28220 Majadahonda, Spain; 15 Department of Medicine, Surgery and Health Sciences, University of Trieste, Strada di Fiume, 447, 34149 Trieste, Italy

**Keywords:** human biomonitoring, children, organophosphate flame retardants, neurodevelopment, WISC, HBM4EU Aligned Studies

## Abstract

The knowledge of the effects of organophosphate flame retardants on children’s neurodevelopment is limited. The purpose of the present research is to evaluate the association between exposure to organophosphate flame retardants and children’s neurodevelopment in two European cohorts involved in the Human Biomonitoring Initiative Aligned Studies. The participants were school-aged children belonging to the Odense Child Cohort (Denmark) and the PCB cohort (Slovakia). In each cohort, the children’s neurodevelopment was assessed through the Full-Scale Intelligence Quotient score of the Wechsler Intelligence Scale for Children, using two different editions. The children’s urine samples, collected at one point in time, were analyzed for several metabolites of organophosphate flame retardants. The association between neurodevelopment and each organophosphate flame retardant metabolite was explored by applying separate multiple linear regressions based on the approach of MM-estimation in each cohort. In the Danish cohort, the mean ± standard deviation for the neurodevelopment score was 98 ± 12; the geometric mean (95% confidence interval (95% CI)) of bis(1,3-dichloro-2-propyl) phosphate (BDCIPP) standardized by creatinine (crt) was 0.52 µg/g crt (95% CI = 0.49; 0.60), while that of diphenyl phosphate (DPHP) standardized by crt was 1.44 µg/g crt (95% CI = 1.31; 1.58). The neurodevelopment score showed a small, negative, statistically imprecise trend with BDCIPP standardized by crt (*β* = −1.30; 95%CI = −2.72; 0.11; *p*-value = 0.07) and no clear association with DPHP standardized by crt (*β* = −0.98; 95%CI = −2.96; 0.99; *p*-value = 0.33). The neurodevelopment score showed a negative trend with BDCIPP (*β* = −1.42; 95% CI = −2.70; −0.06; *p*-value = 0.04) and no clear association with DPHP (*β* = −1.09; 95% CI = −2.87; 0.68; *p*-value = 0.23). In the Slovakian cohort, the mean ± standard deviation for the neurodevelopment score was 81 ± 15; the geometric mean of BDCIPP standardized by crt was 0.18 µg/g crt (95% CI = 0.16; 0.20), while that of DPHP standardized by crt was 2.24 µg/g crt (95% CI = 2.00; 3.52). The association of the neurodevelopment score with BDCIPP standardized by crt was −0.49 (95%CI = −1.85; 0.87; *p*-value = 0.48), and with DPHP standardized by crt it was −0.35 (95%CI = −1.90; 1.20; *p*-value = 0.66). No clear associations were observed between the neurodevelopment score and BDCIPP/DPHP concentrations that were not standardized by crt. No clear associations were observed with bis(1-chloro-2-propyl) phosphate (BCIPP) in either cohort, due to the low detection frequency of this compound. In conclusion, this study provides only limited evidence of an inverse association between neurodevelopment and exposure to BDCIPP and DPHP. The timing of exposure and effect modification of other organophosphate flame retardant metabolites and other substances should be the subject of further investigations that address this scientific hypothesis.

## 1. Introduction

Children’s adequate cognitive development is associated with family members’ educational and socioeconomic attainment and occupational status [1,2], as well as with a home environment that promotes parent–child communication, child autonomy, respect, and caregiving [3]. Exposure to several persistent and non-persistent environmental contaminants may have a negative impact on children’s neurodevelopment [4,5,6,7,8,9,10,11,12]. Flame retardants (FRs) are chemicals that are applied to materials to prevent ignition and the spread of fire. They have been used in many consumer and industrial products since the 1970s [13]. Legacy brominated FRs have been banned under the Stockholm Convention on Persistent Organic Pollutants due to their toxicity and persistence features in the environment and humans [14]. However, the use of their substituents (current-use FRs) is increasing [15,16]. Current-use FRs, like organophosphate FRs (OPFRs), are a class of additive substances, most of which are not restricted for industrial and commercial use in Europe, which raise concerns due to their widespread use to reduce the flammability of commercial products [16], their use as plasticizers [17], and the recently discovered evidence of their toxicity [18,19]. Their possible toxicological properties are receiving greater regulatory attention; in fact, the European Chemicals Agency has classified tris(1,3-dichloro-2-propyl) phosphate (TDCIPP) as a suspected carcinogen [20]. Triphenyl phosphate (TPHP) is currently defined as under assessment as an endocrine disruptor, while tris(1-chloro-2-propyl) phosphate (TCIPP) is not classified [20].

The primary routes of exposure to OPFRs are inhalation and ingestion of house dust and ingestion of food [15,21,22,23]. Exposure to OPFRs in children could be relevant due to their hand-to-mouth behavior [15]. Indeed, higher concentrations of urinary metabolites of OPFRs have been detected among children relative to other age groups [24,25,26,27,28,29,30].

Experimental toxicological analysis for the evaluation of the neurodevelopmental effects of TCIPP, TDCIPP, TCEP, and TPHP has focused primarily on zebrafish, rat brains, and Sebastes rockfish, suggesting possible neurotoxicity due to the modification of several metabolic pathways [31,32,33,34,35,36,37,38].

To date, limited epidemiologic evidence is suggestive of OPFRs’ developmental neurotoxicity [18,39,40,41,42,43]. Most of the evidence derives from epidemiological studies that assessed exposure to OPFRs prenatally using maternal urine samples [39,40,43], while only one study has assessed the link between childhood exposure and cognitive function [42]. Additional research is needed to clarify the full impact of OPFR exposure on the brain during childhood, since the basal structure of the brain is formed mainly during the prenatal period and early childhood [44]. The objective of the present research was to conduct a cross-sectional analysis to evaluate the association between exposure to OPFRs and children’s cognitive performance in two European cohorts of the Human Biomonitoring Initiative (HBM4EU) Aligned Studies [45,46,47]. The Aligned Studies within the HBM4EU Initiative [45,46,48] aim at stimulating capacity building and laying the foundation for a European HBM platform that ensures harmonized data to generate evidence of the actual exposure to chemicals (including OPFRs) and their possible health effects in order to support policymaking.

## 2. Materials and Methods

### 2.1. Data Source

The present research is based on individual data concerning the assessment of child neurodevelopment and the measurement of OPFR exposure in the urine of the Odense Child Cohort (OCC) in Denmark (Odense University Hospital in Denmark) [49,50] and the PCB cohort in Slovakia (Department of Environmental Medicine, Faculty of Public Health, Slovak Medical University) [51,52], which have participated in the HBM4EU Aligned Studies. A detailed description of the sampling methods for inclusion of participants and data harmonization in the HBM4EU Aligned Studies is reported in [45]. In brief, the study participants were 7-year-old children enrolled in the OCC and 11-year-old children belonging to the PCB cohort, for whom the neurodevelopmental evaluation and the measurement of at least one OPFR were available. 

Informed consent was obtained from the caregivers of all participants’ involved in the study. The study was conducted in accordance with the Declaration of Helsinki. The research protocols of the cohorts were approved by their respective ethics committees (PCB cohort: Ethics Committee of the Slovak Medical University from November 18, 2013; OCC: Regional Scientific Ethical Review Committee for Southern Denmark (Project ID S-20090130) and the Danish Data Protection Agency (j.no. 18/33119)).

### 2.2. Study Outcomes

In both cohorts, the Wechsler Intelligence Scale for Children (WISC) [53] was used to assess the neurodevelopment of the children. The 3rd edition of the WISC [54] was used in the PCB cohort, while the 5th edition was used in the OCC [55]. Both editions assess and measure several aspects of the child’s neurodevelopment, providing different index scores for each edition that, when combined, yield the Full-Scale Intelligence Quotient (FSIQ). However, the FSIQ scores obtained from the two editions measure different aspects of children’s neurodevelopment, and they could had not be harmonized. A brief description of the scores obtained from the two editions of the WISC, along with the method of computation, is included in the Supplemental Materials (Table S1).

We considered the FSIQ score, which measures the child’s overall cognitive ability as the main outcome. The FSIQ score ranges from 40 to 160 and is set to have a mean of 100 and a standard deviation of 15 based on Slovak or Danish population-based reference data (standard population). Higher scores should be interpreted as a better test performance and, therefore, better cognitive abilities, although no clinical diagnosis can be made according to any low or very low FSIQ score.

### 2.3. Exposure

In the study cohorts, OPFR exposure was assessed by analyzing the metabolites diphenyl phosphate (DPHP) for TPHP, bis(1,3-dichloro-2-propyl) phosphate (BDCIPP) for TDCIPP, and bis(1-chloro-2-propyl) phosphate (BCIPP) for TCIPP in the children’s urine samples, which were collected concurrently with the neurodevelopment assessment. 

### 2.4. Chemical Analysis

#### 2.4.1. Organophosphate Flame Retardants

Children’ spot urine samples were collected between 2018 and 2019 in the OCC, and between 2014 and 2017 in the PCB cohort [56].

OPFR metabolites in the PCB cohort were analyzed at the RECETOX laboratories, Masaryk University. A modified protocol from Van den Eede et al. [57] was employed for sample preparation. The OPFR metabolites were then analyzed by reverse-phase liquid chromatography, with ammonium fluoride as the mobile-phase modifier, and detected using an electrospray tandem mass spectrometry (ESI-MS/MS) instrument operating in negative mode. Quantitation of the analytes was achieved using the stable isotope dilution method. A full description of the method can be found in the Supplementary Materials.

The OPFR metabolites in the urine samples from the OCC cohort were analyzed at the Toxicological Centre, University of Antwerp, using the method described by Bastiaensen et al. [58]. After solid-phase extraction on Bond-Elut C18 cartridges, the analysis was performed by reverse-phase liquid chromatography (Agilent 1290 Infinity II system and Kinetex biphenyl column) coupled with an ESI-MS/MS instrument operating in negative mode. Quantitation of the analytes was achieved using the stable isotope dilution method. A complete description of the method can be found in the Supplementary Materials. 

Both laboratories that analyzed the urine samples had successfully completed the interlaboratory comparison investigations and external quality assurance schemes, described in detail in [59,60].

#### 2.4.2. Creatinine

To account for urinary dilution, creatinine analysis was performed on the urine samples from the PCB cohort and the OCC, as previously described [56]. In the PCB cohort, the creatinine concentration (µg/L) was determined by flow injection analysis–tandem mass spectrometry (FIA-MS/MS). In the OCC, spectrophotometric determination of creatinine concentrations was conducted on a Konelab 20 Clinical Chemistry Analyzer, using a commercial kit (Thermo, Vantaa, Finland).

### 2.5. Potential Confounders

As described in our previous research [56], based on information obtained from the questionnaires applied in the different HBM4EU Aligned Studies [45,46], the variables were harmonized across the studies.

Potential confounders in neurodevelopment were identified using a literature review and a directed acyclic graph (Supplementary Figure S1) using the tool DAGitty [61]. The available and common potential confounders were the highest education level of the household of the child, body mass index (BMI) z-score, and the sex of the child. The level of education of the parents was based on the International Standard Classification of Education (ISCED) developed by the United Nations Educational, Scientific and Cultural Organization (UNESCO) [62]. Individuals with no to lower secondary education were included in the lower education level (ISCED 0–2), individuals with upper secondary to post-secondary non-tertiary education represented the medium level (ISCED 3–4), and those with tertiary and higher education were included in the high education level (ISCED ≥ 5). The highest education level of the household of the child corresponds to the highest level of education between mother and father.

At the urine sample collection, the children’s height and weight were measured by healthcare staff in each cohort. BMI (kg/m^2^) was calculated as the ratio between the weight (kg) and the height squared (m^2^). BMI was converted into standardized World Health Organization sex- and age-specific z-scores (BMI z-score) [63]. BMI z-scores were categorized as follows: underweight (BMI z-score ≤ −2), normal weight (−2 < BMI z-score ≤ 1), overweight (1 < z-score ≤ 2), and obese (BMI z-score > 2).

The child’s age was not considered as a potential confounder, as the FSIQ score is already weighted for the age at which the WISC is administered, and the children’s urine samples were collected at the same time.

### 2.6. Statistical Analysis

Measurements of BCIPP, BDCIPP, and DPHP were left-censored data, i.e., if a value was below the limit of quantification (LOQ). For measurements below the LOQ, possible values were imputed by using a truncated lognormal distribution. This imputation was conducted for BDCIPP and DPHP, because at least 30% of the values were observed. BCIPP measurements were dichotomized into below/above the LOQ [64], because more than 70% of the observations were below the LOQ [47]. The LOQs of the biomarkers for the OCC samples were 0.1, 0.05, and 0.4 µg/L, while for PCB samples they were 0.09, 0.3, and 0.3 µg/L, for DPHP, BDCIPP, and BCIPP, respectively.

The FSIQ score was obtained from two different editions of the WISC; for this reason, separate statistical analyses were conducted for each cohort. 

In each cohort, the main characteristics of the children and their families, along with the children’s FSIQ scores, were reported as frequencies and percentages for categorical variables or as arithmetic means and standard deviations (SD) for continuous variables. The geometric mean and 95% confidence interval (95% CI), along with the 90th percentile for OPFR biomarkers standardized for creatinine and creatinine, were also calculated. The distributions of the biomarker concentrations are shown in the Supplementary Materials. The frequency and percentage distributions of dichotomized BCIPP were calculated for each cohort. Differences between the two cohorts in the highest education level of the household of the child, and in the mean FSIQ scores, were assessed by the chi-squared test and by applying ANOVA. Furthermore, ANOVA was applied to assess the mean differences in FSIQ scores between the highest education level of the child’s household and the child’s sex for each cohort. Pearson’s correlation was estimated between the BMI z-score and FSIQ score for each cohort.

Simple and multiple linear regressions, adjusted for the highest educational level of the household of the child, along with the child’s sex and BMI z-score, were applied to study the associations between the FSIQ score and each OPFR biomarker. The BDCIPP and DPHP concentrations were standardized according to the child’s urinary creatinine levels (µg/g creatinine) to account for urinary dilution, and then natural logarithm-transformed because of their skewed distribution. BCIPP was included in the models as a dichotomic variable. Separate models were performed for each biomarker in each cohort. Furthermore, separate models were performed for each cohort to study the associations between the FSIQ score and the combined exposure to the three OPFR biomarkers. The robust MM-estimator was used due to violations of the standard ordinary least squares assumptions [65]. Beta (*β*) and the 95% CI of each multiple linear regression, based on the approach of MM-estimation, are presented in the forest plot. 

As sensitivity analyses, simple and multiple regression models based on the approach of MM-estimation were performed, considering the natural logarithm transformation of the OPFR biomarker concentrations without the creatinine standardization. We also performed analyses stratified by (i) the child’s sex, (ii) the highest educational level of the child’s household, and (iii) the categorical BMI z-score to assess whether the effects of natural logarithm transformation of OPFRs standardized for creatinine on neurodevelopment differed between (i) boys and girls, (ii) low, medium, and high levels of education, and (iii) underweight, normal-weight, overweight, and obese subjects.

In simple and multiple regression models based on the approach of MM-estimation, the BDCIPP and DPHP concentrations were also included as categorical variables. BDCIPP and DPHP were categorized using the tertiles of the distribution of the natural logarithm transformation of each biomarker standardized for creatinine in each cohort as cutoffs.

To evaluate the hypothesis that the probability of suboptimal development, measured as a dichotomous variable derived from the distribution of the FSIQ score [66], would depend on the increase in the OPFRs’ concentrations, we also applied logistic regression using the 20th percentile of the FSIQ score in each cohort as the cutoff for suboptimal development.

SAS (version 9.4 SAS Institute Inc., Cary, NC, USA) and R software (package “anthroplus” in R; R version 4.0.5, R Core Team (2021); R: A Language and Environment for Statistical Computing; R Foundation for Statistical Computing, Vienna, Austria; URL https://www.R-project.org/(accessed on 30 July 2023)) were used for the statistical analysis.

## 3. Results

Although 300 children participated in the HBM4EU Aligned Studies for each cohort, only 264 children in the OCC and 297 in the PCB cohort provided information on FSIQ score and at least one OPFR measurement. The child and family characteristics of the OCC and PCB cohorts are shown in Table 1. The mean and SD of the children’s age were 7.0 ± 0.1 in the OCC and 11.1 ± 0.4 in the PCB cohort. The percentage distributions of the highest education level of the household of the child were different between the two cohorts (chi-squared test *p*-value < 0.001): the percentages for medium education were 53.4% and 73.7%, and those of high education were 32.6% and 15.2%, in the OCC and PCB cohorts, respectively. The mean FSIQ score was 98 in the OCC and 81 in the PCB cohort (ANOVA *p*-value < 0.001).

The urinary concentrations of OPFR biomarkers (calculated as µg/g creatinine) and creatinine concentrations for both cohorts are reported in Table 2. The urinary concentrations of OPFR biomarkers for each cohort are reported in Supplementary Table S2. Table 3 reports the frequency and percentage distribution of children regarding BCIPP dichotomized by LOQ (µg/L) in the OCC and the PCB cohort.

As displayed in Table 4, the FSIQ scores increased in each cohort with increasing education level of the household of the child, whereas the mean FSIQ score did not vary by the sex of the child. The Pearson’s correlation between the children’s BMI z-score and FSIQ was weakly positive in the PCB cohort.

**Figure 1:**
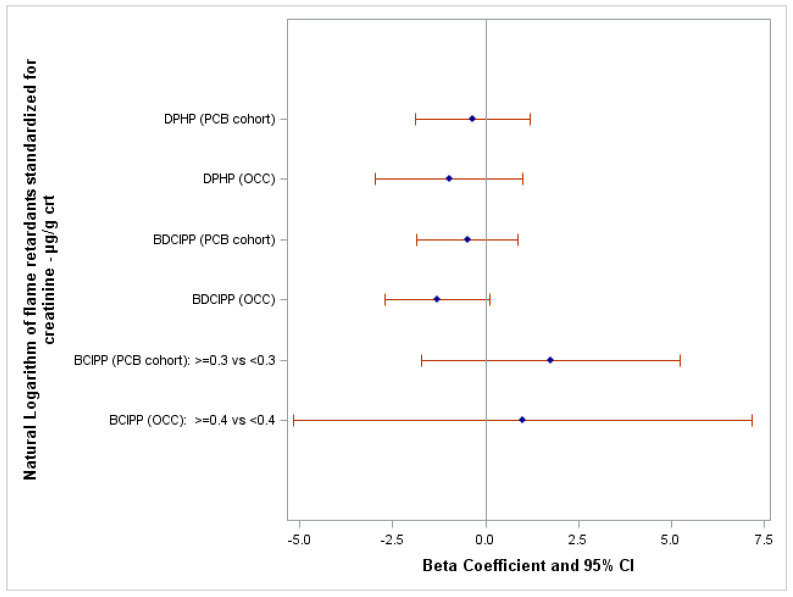
Forest plot showing the association between FSIQ score and the natural logarithm of OPFR biomarkers standardized for creatinine, adjusted for the highest education level of the household of the child, the child’s sex, and the child’s body mass index z-score. Abbreviations: crt, creatinine.

In the OCC, FSIQ scores tended to show a small negative trend, albeit a statistically imprecise one, with BDCIPP (*β* = −1.30; 95%CI = −2.72; 0.11; *p*-value = 0.07), and there was no clear association with DPHP (*β* = −0.98; 95%CI = −2.96; 0.99; *p*-value = 0.33). Among children in the PCB cohort, the association between FSIQ score and BDCIPP (*β* = −0.49; 95%CI = −1.85; 0.87; *p*-value = 0.48) and between FSIQ score and DPHP (*β* = −0.35; 95%CI = −1.90; 1.20; *p*-value = 0.66) was negligible. No associations were observed with BCIPP in either cohort. (Figure 1).

The crude and adjusted β and the 95%CI of multiple linear regression based on the approach of MM-estimation are reported in Supplementary Tables S3 and S4. The crude and adjusted *β* and the 95%CI of multiple linear regression based on the approach of MM-estimation without creatinine standardization are reported in Supplementary Table S5. Supplementary Tables S6–S8 report the results of the simple and adjusted multiple linear regressions based on the approach of MM-estimation stratified by the child’s sex, the highest educational level of the child’s household, and the child’s categorical BMI z-score, respectively. It seems that the effects of natural logarithm transformation of OPFRs standardized for creatinine on neurodevelopment did not differ across the strata. The results of the effects of natural logarithm transformation of OPFRs standardized for creatinine considered as categorical variables (using the tertiles of their distribution in each cohort as cutoffs) on neurodevelopment are included in Supplementary Tables S9 and S10. No clear associations were observed, even when considering exposure to OPFRs as categorical variables.

The results of the simple and multiple linear regression models based on the approach of MM-estimation to study the association between FSIQ scores and the combined exposure to OPFR biomarkers, in each cohort, are shown in Supplementary Tables S11 and S12. No clear associations were observed, even when considering the combined exposure to OPFR biomarkers.

Furthermore, the logistic regressions based on a dichotomous outcome did not show a higher risk of suboptimal development with increasing OPFR concentrations.

## 4. Discussion

### 4.1. Internal Consistency

This cross-sectional study hypothesized that increased levels of biomarkers of OPFRs, adjusted for confounders, might be associated with a concurrent decreased cognitive ability in European children aged 7 and 11 years of age. 

Regarding the PCB cohort, the families came from the Michalovce region, an environmental hotspot where a plant manufacturing polychlorinated biphenyls (PCBs) that operated from 1959 through to the mid-1980s improperly discharged large quantities of PCB-contaminated waste into the surrounding area. Although factory emissions are much more controlled now than in the 1960s and 1970s, and urine samples collected locally between 2014 and 2017 did not show higher OPFR levels than in other European regions [67], exposure to PCB has been associated with lower cognitive performance in some studies [68,69]. The results regarding neurodevelopment and exposure to PCB in the PCB cohort are inconclusive [52,70]. On the other hand, OCC families represent residents of the Odense municipality (Southern Denmark), a city that does not differ greatly from the rest of Denmark in terms of sociodemographic and health indicators [71].

Overall, since the study was based on a relatively small sample size, hence being statistically imprecise and potentially affected by random error, the small negative associations observed between the urinary levels of DPHP (a metabolite of TPHP) and BDCIPP (a metabolite of TDCIPP) and the FSIQ in both cohorts should be interpreted with caution (Figure 1). 

In fact, these results should consider the different contexts of the original cohorts enrolled in the HBM4EU initiative. The two cohorts varied by age (7 years in the OCC vs. 11 years in the PCB cohort), by education level (Table 1; 32.6% in the OCC vs. 15.2% in the PCB cohort, with an ISCED ≥ 5 as the highest education level of the household), by edition of the WISC (the 3rd edition in the PCB cohort and the 5th edition in the OCC), and by FSIQ score (mean scores: 98 in the OCC vs. 81 in the PCB cohort). In terms of concentration, the results showed higher urinary levels of DPHP in the PCB cohort compared to the OCC, both with and without creatinine standardization. Despite this heterogeneity, which might have interfered with the neurodevelopmental score, the *β* scores for FSIQ score were quite similar in the two cohorts. Results pertaining to the BDCIPP metabolite, in both the PCB cohort and the OCC, also showed a reduced FSIQ performance in children with higher exposure, although in the OCC the negative *β* score was lower and nearly reached statistical significance. 

Our study also considered potential confounders of the relationship between OPFR exposure and cognitive neurodevelopment. Consequently, the highest educational level of the household of the child, the child’s sex, and the child’s body mass index z-score were included in the models. Many more factors were considered as potential confounders and then dismissed in the analytical phase, according to an a priori directed acyclic graph (DAG) (Supplementary Figure S1) elaborated during the HBM4EU project [72]. Nevertheless, we cannot exclude the possibility that the effect of residual confounding by unknown factors might be present, involving other determinants of FSIQ that might also be associated with different OPFR exposure levels.

For completeness, we should mention that the final models did not include terms for a direct evaluation of the home environment as designed according to HOME (Home Observation for Measurement of the Environment), which has been found to be associated with neurodevelopment [73]. This variable was not available in the OCC and PCB cohorts. In addition, due to the lack of adequate statistical power, it was beyond the scope of this analysis to evaluate the effects of chemical mixtures. Exposure to mixtures, or to chemical agents other than OPFRs, might have had relevant neurodevelopmental effects, especially in the PCB cohort, which was recruited around the environmental hotspot of Michalovce, Slovakia, where polychlorinated biphenyls, 2,3,7,8-tetrachlorinated dibenzo-dioxin, and dioxin-like toxic equivalents have been the primary objective of epidemiological investigations for many years [70]. For this reason, the inverse association between metabolites of OPFRs and FSIQ performance should be interpreted with caution. 

Furthermore, the error in the *β* scores for OPFRs may have been caused by the absence or inclusion of other variables that were not included in the analyses. 

After modelling, confounding, and effect modification, possible measurement error in the exposure assessment should be included as a possible interpretation of the results. Urinary OPFRs in the two cohorts were measured by two different laboratories, although both laboratories successfully passed the same interlaboratory comparison investigations and external quality assurance schemes. In addition, urinary concentrations of OPFRs can vary over time because of their short biological half-lives [74]; thus, collecting single spot urine samples could potentially lead to exposure misclassification. Future cohort studies should consider collecting multiple samples within the same day to improve the exposure characterization.

### 4.2. External Consistency

The scientific evidence concerning the relationship between exposure to OPFRs (specifically BCIPP, BDCIPP, and DPHP) and the neurological and behavioral development of children aged 7–11 years is sparse, due to the relatively recent introduction of OPFRs in the market following the gradual phase-out of some brominated flame retardants [14]. Unlike our cross-sectional design, most of the external evidence comes from epidemiological studies that considered exposure to OPFRs in the prenatal phase by analyzing maternal urine samples and subsequently assessed neurodevelopment at various ages during childhood using different tests or different editions of the same test.

Firstly, the CHAMACOS study, conducted between October 1999 and October 2000 in a group of low-income pregnant women of Hispanic ethnicity located in California, involved an assessment of the cognitive performance of 310 children aged 7 years [39]. The children’s FSIQ was measured according to the WISC-IV. A proxy of child prenatal exposure to OPFRs had been determined from the concentrations of BDCIPP and DPHP in the urinary sample obtained from the child’s mother during pregnancy. The median urinary level of BDCIPP among mothers in the CHAMACOS cohort was 0.4 ng/mL; the corresponding level of DPHP was 0.9 ng/mL. These values do not differ greatly from the urinary levels of children aged 7–11 in our analyses. In the CHAMACOS cohort, a borderline statistically significant decrease in FSIQ with increasing prenatal DPHP was observed in multivariable regression models adjusted for important confounders. Specifically, for each 10-fold increase in prenatal urinary DPHP concentration, the FSIQ was reduced by 2.9 points (95%CI: −6.3; 0.5). In contrast, no significant association was found between neurodevelopment and BDCIPP. In this case, for each 10-fold increase in prenatal urinary BDCIPP concentration, the FSIQ was increased by 0.2 points (95%CI: −1.9; 2.4). 

A second American study from Ohio assessed the maternal prenatal exposure to a mixture of OPFR metabolites, including BDCIPP and DPHP, and prospectively evaluated children’s FSIQ (WISC-IV) at 8 years of age [43]. The median exposure levels at delivery were 0.66 and 1.69 ng/mL for BDCIPP and DPHP, respectively. Neither BDCIPP nor DPHP levels in urine during pregnancy were associated with FSIQ in the Ohio study after adjusting for potential confounding variables. 

A further analysis was carried out in the same cohort, evaluating the exposure of children to BDCIPP, BCEP, and DPHP at 1, 2, 3, and 5 years of age, while cognitive abilities were assessed using the WISC-IV neurodevelopmental test at 8 years. The association between IQ and exposure to BCEP and BDCIPP tended to be positive, while the association between IQ and exposure to DPHP tended to be inverse [42].

Furthermore, an analysis was conducted on the association between prenatal exposure to DPHP and BDCIPP and executive function in 340 Norwegian preschoolers from the Norwegian Mother, Father, and Child Cohort Study [40]. In this case, higher prenatal concentrations of DPHP and BDCIPP were associated with lower Stanford–Binet (SB)5 verbal working memory [40].

## 5. Conclusions

The recent publication of policy briefs by the Human Biomonitoring for Europe project (https://www.hbm4eu.eu/wp-content/uploads/2022/06/HBM4EU_Policy-Brief-FlameRetardants.pdf accessed on 1 July 2023) highlights how the potential exposure of children to currently used FRs is of particular concern among European citizens. However, concurrently, adequate monitoring of OPFR metabolites among representative samples of European children has been conducted in only a few countries. As a consequence, our investigation was also restricted to two cohorts, who had quite heterogeneous distribution of exposure and outcomes. This makes it difficult to generalize our conclusions. In addition, while the literature that considers a range of neurodevelopmental effects of exposure to OPFRs remains rather inconsistent, our aligned analysis of two groups of European children also displayed no clear associations between FSIQ and exposure to BDCIPP and DPHP. The timing of exposure and the effect modification of other OPFR metabolites and other substances should be the subject of further investigations that address this scientific hypothesis. A more extensive, prospective use of human biomonitoring for risk assessment and policy on a complete set of OPFRs that are potentially present in the general population could resolve some of the limitations in our current understanding of OPFRs’ health effects. 

## Figures and Tables

**Table 1:** Children’s and their families’ characteristics in the OCC and the PCB cohort.

Characteristics	OCC	PCB Cohort
**Child’s sex, N (%):**		
Male	142 (53.8)	131 (44.1)
Female	122 (46.2)	166 (55.9)
**Highest education level of the household of the child, N (%):**		
Low education (ISCED 0–2)	37 (14.0)	16 (5.4)
Medium education (ISCED 3–4)	141 (53.4)	219 (73.7)
High education (ISCED ≥ 5)	86 (32.6)	45 (15.2)
Missing	0 (0.0)	17 (5.7)
**Child’s age, mean ± SD (N):**	7.0 ± 0.1 (264)	11.1 ± 0.4 (297)
**Child’s BMI z-score, mean ± SD (N):**	−0.1 ± 1.0 (259)	0.7 ± 1.3 (297)
**FSIQ score, mean ± SD (N):**	98 ± 12 (264)	81 ± 15 (297)

Abbreviations: OCC, Odense Child Cohort; ISCED, International Standard Classification of Education; SD, standard deviation; BMI, body mass index.

**Table 2:** Urinary concentrations of creatinine and OPFR biomarkers standardized for creatinine in the OCC and the PCB cohort.

Biomarkers of Exposure	N	Geometric Mean (95%CI)	25th Percentile	Median	75th Percentile	90th Percentile
OPFRs (µg/g crt)						
DPHP:						
OCC	264	1.44 (1.31; 1.58)	0.86	1.39	2.34	3.82
PCB cohort	296	2.24 (2.00; 2.52)	1.32	2.21	3.45	5.06
BDCIPP:						
OCC	264	0.52 (0.49; 0.60)	0.25	0.49	0.92	2.21
PCB cohort	296	0.18 (0.16; 0.20)	0.08	0.17	0.35	10.73
Crt (g/L)						
OCC	264	0.70 (0.65; 0.75)	0.51	0.78	1.05	1.32
PCB cohort	296	1.21 (1.12; 1.31)	0.88	1.31	1.82	2.39

Abbreviations: 95%CI, 95% confidence interval; BDCIPP, bis(1,3-dichloro-2-propyl) phosphate; DPHP, diphenyl phosphate, Crt, creatinine.

**Table 3:** Frequency and percentage distribution of children regarding BCIPP dichotomized by LOQ (µg/L) in the OCC and the PCB cohort.

BCIPP µg/L	N (%)
OCC:	
<0.4	247 (93.6)
≥0.4	17 (6.4)
PCB cohort:	
<0.3	210 (70.7)
≥0.3	87 (29.3)

Abbreviations: BCIPP, bis(1-chloro-2-propyl) phosphate.

**Table 4:** Mean ± SD of FSIQ scores by characteristics of the OCC and the PCB cohort.

	FSIQ			
Characteristics	OCC	*p*-Value	PCB Cohort	*p*-Value
**Highest education level of the household of the child,** **mean ± SD** **(N):**				
Low education (ISCED 0–2)	95 ± 14 (38)	0.20 ^a^	56 ± 9 (16)	<0.01 ^a^
Medium education (ISCED 3–4)	98 ± 12 (141)		81 ± 13 (219)	
High education (ISCED ≥5)	99 ± 11 (86)		91 ± 16 (45)	
**Child’s sex, mean ± SD** **(N)** **:**				
Male	97 ± 12 (142)	0.07 ^a^	79 ± 16 (131)	0.09 ^a^
Female	100 ± 12 (122)		82 ± 15 (166)	
**Child’s BMI z-score, correlation (N):**	0.03 (259)	0.66 ^b^	0.16 (297)	0.01 ^b^

^a^ ANOVA; ^b^ Pearson’s correlation test.

## Data Availability

Metadata of the three cohorts are available from IPCHEM, the European Commission’s Information Platform for Chemical Monitoring. The summary statistics (percentiles P5, P10, P25, P50, P75, P90, P95) of the exposure biomarkers will be made available on the openly accessible online European HBM dashboard (https://www.hbm4eu.eu/eu-hbm-dashboard/ accessed on 30 June 2023) and IPCHEM (https://ipchem.jrc.ec.europa.eu/ accessed on 30 June 2023), where they can be accessed. Data sharing of individual-level data is possible upon request.

## Supplementary Materials

The following supporting information can be downloaded at: https://www.mdpi.com/article/10.3390/toxics11110878/s1, Table S1: List of scores (variables) from the WISC-III and WISC-V; Table S2: Urinary concentrations (µg/L) of OPFR biomarkers in the OCC and the PCB cohort; Table S3: Simple and multiple linear regression models based on the approach of MM-estimation between the FSIQ score and the natural logarithm transformation of OPFR biomarkers standardized for creatinine in the OCC and the PCB cohort; Table S4: Simple and multiple linear regression models based on the approach of MM-estimation between the FSIQ score and BCIPP dichotomized by LOQ in the OCC and the PCB cohort; Table S5: Simple and multiple linear regression models based on the approach of MM-estimation between the FSIQ score and the natural logarithm transformation of OPFR biomarkers in the OCC and the PCB cohort; Table S6: Simple and multiple linear regression models based on the approach of MM-estimation between the FSIQ score and the natural logarithm transformation of OPFR biomarkers standardized for creatinine, and between the FSIQ score and binary OPFR biomarkers, in the OCC and the PCB cohort, stratified by gender; Table S7: Simple and multiple linear regression models based on the approach of MM-estimation between the FSIQ score and the natural logarithm transformation of OPFR biomarkers standardized for creatinine, and between the FSIQ score and binary OPFR biomarkers, in the OCC and the PCB cohort, stratified by the highest level of education of the child’s household; Table S8: Simple and multiple linear regression models based on the approach of MM-estimation between the FSIQ score and the natural logarithm transformation of OPFR biomarkers standardized for creatinine, and between the FSIQ score and binary OPFR biomarkers, in the OCC and the PCB cohort, stratified by the categorical BMI z-score; Table S9: Simple and multiple linear regression models based on the approach of MM-estimation between the FSIQ score and the natural logarithm transformation of OPFR biomarkers standardized for creatinine, categorized by tertiles, in the whole OCC and stratified by the child’s sex; Table S10: Simple and multiple linear regression models based on the approach of MM-estimation between the FSIQ score and the natural logarithm transformation of OPFR biomarkers standardized for creatinine, categorized by tertiles, in the whole PCB cohort and stratified by the child’s sex; Figure S1: Directed acyclic graph (DAG) representing the assumed relationships between the exposure to OPFRs and the neurodevelopmental outcomes, specific for children. Table S11: Simple and multiple linear regression models based on the approach of MM-estimation between the FSIQ score and natural logarithm transformation of OPFRs biomarkers standardized for creatinine and binary OPFRs biomarkers in the OCC and PCB cohort, Table S12: Simple and multiple linear regression models based on the approach of MM-estimation between the FSIQ score and natural logarithm transformation of OPFRs biomarkers and binary OPFRs biomarkers in the OCC and PCB cohort [75,76,77].
