# Supplementary material for: Cognitive Performance and Exposure to Organophosphate Flame Retardants in Children: Evidence from a Cross-Sectional Analysis of Two European Mother–Child Cohorts
Source: Toxics. 2023 Oct 24;11(11):878. doi: 10.3390/toxics11110878 (PMC10675051; doi:10.3390/toxics11110878)
Supplement: Supplementary file 1 [file toxics-11-00878-s001.zip › toxics-2643377-supplementary.pdf]

Supplementary Materials

Cognitive Performance and Exposure to Organophosphate Flame Retardants in Children: Evidence from a Cross-Sectional Analysis of Two European Mother–Child Cohorts

Valentina Rosolen, Elisa Giordani, Marika Mariuz, Maria Parpinel, Vicente Mustieles, Liese Gilles, Eva Govarts, Laura Rodriguez Martin, Kirsten Baken, Greet Schoeters, Ovnair Sepai, Eva Sovcikova, Lucia Fabelova, Jiři Kohoutek, Tina Kold Jensen, Adrian Covaci, Maarten Roggeman, Lisa Melymuk, Jana Klánová, Argelia Castano, Marta Esteban López and Fabio Barbone

Description of organophosphate flame retardants chemical analysis

PCB cohort (Slovakia)

Organophosphate flame retardants (OPFR) metabolites in the PCB cohort were analyzed at the RECETOX Laboratories, Masaryk University. Samples were analyzed by liquid chromatography electrospray ionization mass spectrometry using an Agilent 1200 series liquid chromatography (HPLC) system. Chromatographic separation was accomplished using a Waters Acquity BEH C18 analytical column (100 x 2.1 mm, 1.7 µm particle size) maintained at 30°C and equipped with Acquity BEH C18 VanGuard pre-column. The mobile phases for the gradient separation of the analytes were 1.0 mM water solution of ammonium fluoride (component A) and methanol with an addition of 1.0 mM ammonium fluoride (component B). The flow rate was 0.3 mL/min, and the injection volume was 5 µL. Analyte detection was performed by a tandem mass spectrometer AB Sciex QTrap 5500 operating in negative electrospray ionization mode at 450°C with N2 as a nebulizer gas and a capillary voltage of -4kV. Deuterium-labeled bis(1-chloro-2-propyl) phosphate (BCIPP), bis(1,3-dichloro-2-propyl) phosphate (BDCIPP) and diphenyl phosphate (DPHP) isotope dilution method was used for quantification of the analytes. The linear quantification range (MRM mode) was 0.1-100 µg/L urine, with limits of quantification from 0.09 to 1.0 µg/L urine for respective analytes (MQL, calculated as 10* the standard deviation (SD) of the blank sample set concentration).

Each batch of samples contains blank samples (matrix), reference material (pooled sample with known concentrations of analytes obtained during HBM4EU intercalibration study) and spiked in-house QC sample (urine spiked with known concentration of analytes. Respective numbers of QC samples are given in table below.

OCC cohort (Denmark)

OPFR metabolites in the OCC cohort were analyzed at the Toxicological Center, University of Antwerp. A volume of 1.5 mL of urine was added to a clean tube together with deuterium-labelled internal standards (IS, 5 ng), 1 mL phosphate buffer (1 M, pH 6) and 50 µL β-glucuronidase enzyme solution (E. coli, 2 mg/mL in phosphate buffer, pH 6). After 2 h incubation at 37 °C, target analytes were acidified with 100 µL formic acid, extracted by solid phase extraction on Bond-Elut C18 cartridges (3 mL, 200 mg, Agilent Technologies, Santa Clara, CA, USA) and eluted using 3 mL methanol. Final extracts were reconstituted in 100 µL of ultrapure water:methanol (50/50, v/v) and passed through 0.2 µm nylon centrifugal filters (VWR International, Leuven, Belgium) before analysis.

OPFR metabolites were separated on a Kinetex Biphenyl reversed-phase column (2.1 × 100 mm, 2.6 µm; Phenomenex, Torrance, CA, USA) and detected using an Agilent 1290 Infinity II liquid chromatography system coupled to a 6495 triple quadrupole mass spectrometer (Agilent Technologies, Santa Clara, CA,

USA). Mobile phases consisted of ultrapure water (UPW) with 2% methanol (A) and methanol with 2% ultrapure water (B), both with 5 mM ammonium acetate.

Neat solvent calibration curves were used to quantify PFR metabolite concentrations in urine samples, ranging from 0.4 to 300 ng/mL for BCIPP, and from 0.08 to 60 ng/mL for DPHP and BDCIPP. Limits of quantification (LOQ) of PFR metabolites are 0.1, 0.4 and 0.05 ng/mL for DPHP, BCIPP and BDCIPP respectively. LOQs were calculated as three times the standard deviation of the procedural blank concentrations for compounds which are present in blanks or as the concentration corresponding to a signal-to-noise ratio of 10 in low level quality control samples. Concentrations of OPFR metabolites in the procedural blanks were subtracted from the levels in urine.

Several quality control (QC) measures were undertaken to guarantee the validity of the obtained results. Procedural blank samples (UPW extraction) and quality control samples (UPW with native standards spiked at two levels – low and medium) were analysed in parallel to the urine samples (two blanks, one QC-low and one QC-medium sample per 40 samples). Results of the internal quality assurance showed a good accuracy for the QC samples (accuracy ranged between 82 and 105% for the three investigated compounds) and a good precision for the replicate QC samples (RSD < 14%). Additional external quality control was assured by successful participation in inter-laboratory comparison exercises such as the Human Biomonitoring for Europe External Quality Assurance Scheme (HBM4EU ICI/EQUAS, 2018–2020 and the External Quality Assessment Scheme for Organic Substances in urine (OSEQAS, 2018–2021).

Description of WISC-III and WISC-V

The two editions of the Wechsler Intelligence Scale of Children (WISC) test assess and measure different aspects of the child's neurodevelopment. The core subtest and the index scores of each edition are listed in **Table S1**

Table S1. List of scores (variables) from WISC-III and WISC-V.

WISC-III		WISC-V	
**CORE SUBTEST**	**Min-Max**	**CORE SUBTEST**	**Min-Max**
Similarities	1-19	Similarities	1-19
Comprehension	1-19		1-19
Vocabulary	1-19	Vocabulary	1-19
Arithmetic	1-19		1-19
Information	1-19		
Block Design	1-19	Block Design	1-19
Picture Completion	1-19	Visual Puzzles	1-19
Coding	1-19		
Object Assembly	1-19		
Picture Arrangement	1-19		
		Digit Span	1-19
		Picture Span	1-19
		Coding	1-19
		Symbol Search	1-19
		Matrix reasoning	1-19
		Figure Weights	1-19
INDEX SCORES	Min-Max	INDEX SCORES	Min-Max
Verbal IQ (VIQ)	40-160	Verbal Comprehension Index (VCI)	40-160
Performance IQ (PIQ)	40-160	Visual Spatial Index (VSI)	40-160
		Fluid Reasoning Index (FRI)	40-160
		Working Memory Index (WMI)	40-160
		Processing Speed Index (PSI)	40-160
Full Scale IQ (FSIQ)	40-160	Full Scale IQ (FSIQ)	40-160

The WISC-III edition[74, 75] comprises 10 core subtests that compute Verbal IQ (VIQ) and Performance IQ (PIQ), which, when combined, yield the Full-Scale IQ (FSIQ) (Mean = 100 and Standard Deviation = 15). The Verbal scale includes Vocabulary, Similarities, Comprehension, Arithmetic, and Digit Span subtests, whereas the performance scale includes Block Design, Picture Arrangement, Picture Completion, Object Assembly, and Coding subtests for all subtests (mean = 10 and standard deviation = 3).

The WISC-V [76] comprises 10 core subtests, yielding five index scores that combine into the FSIQ. The Verbal Comprehension Index (VCI), the Visual Spatial Index (VSI), the Fluid Reasoning Index (FRI), the Working Memory Index (WMI), and the Processing Speed Index (PSI). The FSIQ is derived from 7 of the 10 primary subtests:

- the Verbal Comprehension Index is derived from the Similarities and Vocabulary subtests;
- the Visual Spatial Index (VSI) is derived from the Block Design and Visual Puzzles subtests;
- the Fluid Reasoning Index (FRI) is derived from the Matrix Reasoning and Figure Weights subtests;
- the Working Memory Index is derived from the Digit Span and Picture Span subtests;
- the Processing Speed Index is derived from the Coding and Symbol Search subtests;
- Index standard scores have a mean of 100 and a standard deviation of 15.

Computation of the Core Subtests and Index Scores

The first step is to sum the individual item scores to obtain the raw scores according to the instructions present in the WISC manual. To obtain the core subtests, the sum of raw scores are weighted for the child's age at which the test was administered using the conversion tables (present in the manual of the WISC), which are different for each country and in each WISC edition. The second step is to sum the core subtests, according to the instructions present in the WISC manual, and transform these sums into intelligence quotient based on the population-based reference data (standard population) with a mean of 100 and standard deviation of 15. Each WISC edition in each country has an excellent standardization sample (more than 2000 children ages 6:0 to 16:11) that closely represents the population of each country on stratification variables of race, ethnicity, geographic region, parent education level, and gender.

74. Nader, A.M.; Jelenic, P.; Soulières, I. Discrepancy between WISC-III and WISC-IV Cognitive Profile in Autism Spectrum: What Does It Reveal about Autistic Cognition? *PLoS ONE* **2015**, *10*, e0144645. https://doi.org/10.1371/journal.pone.0144645.
75. Wechsler, D. *Wechsler Intelligence Scale for Children*, 3rd ed.; Canadian (WISC-III); Psychological Corporation. ed.: Toronto, ON, Canada, 1991.
76. Olivier, T.W., Mahone, E.M., Jacobson, L.A. Wechsler Intelligence Scale for Children. In *Encyclopedia of Clinical Neuropsychology*; Kreutzer, J.S., DeLuca, J., Caplan, B., Eds.; Springer: Cham, Switzerland, 2018. https://doi.org/10.1007/978-3-319-57111-9_1605.

Table S2. Urinary concentrations (μg/L) of OPFR biomarkers in the OCC and PCB cohort.

OPFR of biomarker	N	Geometric mean (95%CI)	25th percentile	Median	75th percentile	90th percentile
DPHP:						
OCC	264	1.00 (0.90, 1.12)	0.55	1.02	1.76	3.16
PCB cohort	297	2.72 (2.43, 3.04)	1.50	3.01	4.72	7.74
BDCIPP:						
OCC	264	0.37 (0.32, 0.42)	0.18	0.35	0.66	1.61
PCB cohort	297	0.22 (0.19, 0.24)	0.11	0.21	0.42	0.87

Abbreviations: 95%CI, 95% confidence interval; BDCIPP, bis(1,3-dichloro-2-propyl) phosphate; DPHP, diphenyl phosphate.

Table S3. Simple and multiple linear regression models based on the approach of MM-estimation between the FSIQ score and the natural logarithm transformation of OPFRs biomarkers standardized for creatinine in the OCC and PCB cohort.

	OCC		PCB cohort	
Biomarker of exposure	Simple regression (MM-estimation)	Multiple regression (MM-estimation)†	Simple regression (MM-estimation)	Multiple regression (MM-estimation)†
	β (95%CI); N	β (95%CI); N	β (95%CI); N	β (95%CI); N
OPFR **(μg/g crt):**				
DPHP	-1.73 (-3.72; 0.26)*; 264	-0.98 (-2.96; 0.99); 259	-0.32 (-2.12; 1.48); 296	-0.35 (-1.90; 1.20); 279
BDCIPP	-1.64 (-3.06; -0.21)**; 264	-1.30 (-2.72; 0.11)*; 259	-0.44 (-2.02; 1.15); 296	-0.49 (-1.85; 0.87); 279

Abbreviations: FSIQ, Full Scale Intelligence Quotient; β, beta coefficient. †Adjusted for children's sex and body mass index z-score and the highest level of education of the household of the child. **p-value≤0.05; *p-value≤0.10

Table S4. Simple and multiple linear regression models based on the approach of MM-estimation between the FSIQ score and the BCIPP dichotomized by LOQ, in the OCC and PCB cohort.

	OCC		PCB cohort	
Biomarker of exposure	Simple regression (MM-estimation)	Multiple regression (MM-estimation)†	Simple regression (MM-estimation)	Multiple regression (MM-estimation)†
	β (95%CI); N	β (95%CI); N	β (95%CI); N	β (95%CI); N
**BCIPP (μg/g):**				
LOQ≥0.3 vs LOQ<0.3	-	-	0.79 (-3.30; 4.89); 297	1.77 (-1.72; 5.25); 280
LOQ≥0.4 vs LOQ<0.4	0.96 (-5.20; 7.13); 264	0.99 (-5.19; 7.16); 259	-	-

Abbreviations: FSIQ, Full Scale Intelligence Quotient; β, beta coefficient. †Adjusted for children's sex and body mass index z-score and the highest level of education of the household of the child; BCIPP, bis(1-chloro-2-propyl) phosphate. **p-value≤0.05; *p-value≤0.10

Table S5. Simple and multiple linear regression models based on the approach of MM-estimation between the FSIQ score and the natural logarithm transformation of OPFRs biomarkers in the OCC and PCB cohort.

	OCC		PCB cohort	
Biomarker of exposure	Simple regression (MM-estimation)	Multiple regression (MM-estimation)†	Simple regression (MM-estimation)	Multiple regression (MM-estimation)†
	β (95%CI); N	β (95%CI); N	β (95%CI); N	β (95%CI); N
**OPFR (μg/L)**				
DPHP	-1.58 (-3.35; 0.21)*; 264	-1.09 (-2.87; 0.68); 259	0.01 (-1.89; 1.92); 297	-0.18 (-1.77; 1.41); 280
BDCIPP	-1.72 (-3.09; -0.34)**; 264	-1.42 (-2.79; -0.06)**; 259	-0.13 (-1.81; 1.54); 297	-0.30 (-1.72; 1.11); 280

† Adjusted for children's sex and body mass index z-score and the highest level of education of the household of the child. **p-value≤0.05; *p-value≤0.10

Table S6. Simple and multiple linear regression models based on the approach of MM-estimation between the FSIQ score and the natural logarithm transformation of OPFRs biomarkers standardized for creatinine and between FSIQ score and binary OPFRS biomarkers in the OCC and PCB cohort, stratified by gender.

		OCC		PCB cohort	
		**Simple regression (MM-estimation)**	**Multiple regression (MM-estimation)†**	**Simple regression (MM-estimation)**	**Multiple regression (MM-estimation)†**
**Biomarker exposure**	**Child's sex:**	**β (95%CI); N**	**β (95%CI); N**	**β (95%CI); N**	**β (95%CI); N**
**OPFR (μg/g crt):**					
DPHP	Females	-1.85 (-5.3; 1.43); 122	-1.35 (-4.77; 2.07); 120	-0.48 (-3.07; 2.11); 166	-0.48 (-2.85; 1.90); 160
	Males	-1.28 (-3.78; 1.22); 142	-0.82 (-3.26; 1.62); 139	0.03 (-2.49; 2.55); 130	0.11 (-1.99; 2.21); 119
BDCIPP	Females	-3.23 (-5.45; -1.01)**; 122	-3.25 (-5.51; -0.99)**; 120	-0.66 (-2.73; 1.41); 166	-0.88 (-2.75; 0.99); 160
	Males	-0.24 (-2.05; 1.57); 142	0.05 (-1.59; 2.67); 139	0.16 (-2.24; 2.55); 130	0.02 (-1.96; 2.01); 119
**Binary OPFRs μg/g:**					
BCIPP≥0.4 **VS** BCIPP<0.4	Females	0.27 (-8.44; 8.98); 122	1.71 (-7.56; 10.99); 120	-	-
	Males	1.43 (-7.19; 10.06); 142	0.31 (-8.18; 8.80); 139	-	-
BCIPP≥0.3 **VS** BCIPP<0.3	Females	-	-	-1.10 (-6.19; 4.00); 166	-0.08 (-4.73; 4.56); 160
	Males	-	-	3.29 (-3.11; 9.69); 131	3.22 (-2.19; 8.64); 120

Abbreviations: FSIQ, Full Scale Intelligence Quotient; β, beta coefficient. †Adjusted for children's body mass index z-score and the highest level of education of the household of the child.
**p-value≤0.05; *p-value≤0.10

Table S7. Simple and multiple linear regression models based on the approach of MM-estimation between the FSIQ score and the natural logarithm transformation of OPFRs biomarkers standardized for creatinine and between FSIQ score and binary OPFRS biomarkers in the OCC and PCB cohort, stratified by the highest level of education of the household of the child.

**Biomarkers exposure**	**Highest level of education of the household of the child[a]:**	**OCC**		**PCB cohort**	
		**Simple regression (MM-estimation)**	**Multiple regression (MM-estimation)†**	**Simple regression (MM-estimation)**	**Multiple regression (MM-estimation)†**
		**β (95%CI); N**	**β (95%CI); N**	**β (95%CI); N**	**β (95%CI); N**
**OPFRs (µg/g crt):**					
DPHP	Low education	2.10 (-3.81; 8.01); 37	2.00 (-3.92; 7.92); 37	0.76 (-6.14; 7.65); 16	4.81 (-1.87; 11.49); 16
	Medium education	-2.66 (-5.38; 0.05)*; 141	-2.18 (-4.84; 0.48); 137	0.65 (-1.10; 2.39); 218	0.72 (-0.97; 2.41); 218
	High education	-0.65 (-3.99; 2.70) ; 86	-0.62 (-4.03; 2.78); 85	-3.45 (-7.49; 0.58)*; 45	-2.96 (-7.18; 1.25); 45
BDCIPP	Low education	2.13 (-1.71; 5.98); 37	2.11 (-1.74; 5.96); 37	0.81 (-5.12; 6.74); 16	-0.04 (-7.06; 6.98); 16
	Medium education	-2.31 (-4.29; -0.33)**; 141	-2.00 (-3.95; -0.04)**; 137	-0.30 (-1.85; 1.25); 218	-0.55 (-2.07; 0.96); 218
	High education	-2.26 (-4.59; 0.07)*; 86	-2.27 (-4.59; 0.05)*; 85	0.74 (-3.26; 4.74); 45	0.47 (-3.44; 4.39); 45
**Binary OPFRs (µg/g):**					
BCIPP≥0.4 **VS** BCIPP<0.4	Low education	*Not estimated because all the children with low education had a BCIPP<0.4*	*Not estimated because all the children with low education had a BCIPP<0.4*	-	-
	Medium education	-0.01 (-8.37; 8.36); 141	1.58 (-6.76; 9.93); 137	-	-
	High education	0.50 (-8.39; 9.39); 86	0.14 (-8.58; 8.86); 85	-	-
BCIPP≥0.3 **VS** BCIPP<0.3	Low education	-	-	9.23 (-2.10; 20.55); 16	11.24 (-1.67; 24.14); 16
	Medium education	-	-	0.80 (-3.14; 4.74); 219	0.78 (-3.00; 4.56); 219
	High education	-	-	4.93 (-6.74; 16.60); 45	2.52 (-9.24; 14.28); 45

Abbreviations: FSIQ, Full Scale Intelligence Quotient; [a]Low education (ISCED 0-2), Medium education (ISCED 3-4), High education (ISCED ≥5); β, beta coefficient. †Adjusted for children's sex and body mass index z-score. **p-value≤0.05; *p-value≤0.10

Table S8. Simple and multiple linear regression models based on the approach of MM-estimation between the FSIQ score and the natural logarithm transformation of OPFRs biomarkers standardized for creatinine and between FSIQ score and binary OPFRS biomarkers in the OCC and PCB cohort, stratified by the bmi z-score in categories.

		**OCC**		**PCB cohort**	
		**Simple regression (MM-estimation)**	**Multiple regression (MM-estimation)†**	**Simple regression (MM-estimation)**	**Multiple regression (MM-estimation)†**
**OPFRs biomarkers:**	**Body Mass Index z-score[a]:**	**β (95%CI); N**	**β (95%CI); N**	**β (95%CI); N**	**β (95%CI); N**
**OPFRs (µg/g crt):**					
DPHP	Underweight	-	-	*4 children, not estimated*	*4 children, not estimated*
	Normal weight	-1.62 (-3.73; 0.49); 223	-1.23 (-3.31; 0.85); 223	-0.19 (-2.33; 1.94); 174	-0.26 (-2.08; 1.56); 161
	Overweight	-1.59 (-8.76; 5.57); 25	-3.24 (-12.13; 5.65); 25	0.42 (-5.91; 6.74); 65	-0.24 (-6.39; 5.92); 64
	Obese	4.52 (-9.02; 18.07); 9	8.80 (-2.69; 20.29); 9	-0.46 (-4.36; 3.43); 53	0.92 (-2.35; 4.18); 50
BDCIPP	Underweight	-	-	*4 children, not estimated*	*4 children, not estimated*
	Normal weight	-1.84 (-3.37; -0.31)**; 223	-1.69 (-3.21; -0.17)** ; 223	-0.15 (-2.24; 1.94); 174	0.17 (-1.59; 1.93); 161
	Overweight	-1.52 (-5.72; 2.68); 25	-1.93 (-6.40; 2.54); 25	-2.39 (-5.50; 0.71); 65	-2.60 (-5.34; 0.43)*; 64
	Obese	1.19 (-8.90; 11.27); 9	2.16 (-6.88; 11.19); 9	1.20 (-2.41; 4.82); 53	2.08 (-0.98; 5.15); 50
**Binary OPFRs (µg/g):**					
BCIPP≥0.4 **VS** BCIPP<0.4	Underweight	*2 children, not estimated*	*2 children, not estimated*	-	-
	Normal weight	1.03 (-5.62; 7.69); 223	0.48 (-6.10; 7.06); 223	-	-
	Overweight	10.46 (-11.65; 32.57); 25	*Not estimated*	-	-
	Obese	2.88 (-27.29; 33.05); 9	*Not estimated*	-	-
BCIPP≥0.3 **VS** BCIPP<0.3	Underweight	-	-	*4 children, not estimated*	*4 children, not estimated*
	Normal weight	-	-	2.10 (-3.22; 7.41); 174	2.80 (-1.67; 7.26); 161
	Overweight	-	-	-4.30 (-14.23; 5.64); 66	-6.87 (-16.57; 2.84); 65
	Obese	-	-	0.53 (-7.82; 8.88); 53	1.27 (-6.05; 8.58); 50

Abbreviations: FSIQ, Full Scale Intelligence Quotient;[a] Underweight (z-score≤-2), Normal weight (-2<z-score≤1), Overweight (1<z-score≤2) and Obese (z-score>2); β, beta coefficient. †Adjusted for children's sex and the highest level of education of the household of the child. **p-value≤0.05; *p-value≤0.10

Table S9. Simple and multiple linear regression models based on the approach of MM-estimation between the FSIQ score and the natural logarithm transformation of OPFRs biomarkers standardized for creatinine categorized by tertiles, in the whole OCC and stratified by child's sex.

	**OCC**	
	**Simple regression (MM-estimation)**	**Multiple regression (MM-estimation)†**
**OPFRs biomarkers:**	**β (95%CI)**	**β (95%CI)**
**DPHP (µg/g crt):**	***N=264***	***N=259***
-0.15 ≤ DPHP < 0.66 **VS** DPHP < -0.15	-0.69 (-4.51; 3.13)	-0.05 (-3.81; 3.72)
DPHP ≥ 0.66 **VS** DPHP < -0.15	-3.37 (-7.22; 0.48)*	-2.18 (-6.01; 1.65)
**BDCIPP (µg/g crt):**	***N=264***	***N=259***
-1.14 ≤ BDCIPP < -0.31 **VS** BDCIPP < -1.14	-0.95 (-4.83; 2.93)	0.16 (-3.67; 3.99)
BDCIPP ≥ -0.31 **VS** BDCIPP < -1.14	-3.04 (-6.90; 0.83)	-2.44 (-6.23; 1.34)

Abbreviations: FSIQ, Full Scale Intelligence Quotient; β, beta coefficient. †Adjusted for children's sex and body mass index z-score, and the highest level of education of the household of the child.
**p-value≤0.05; *p-value≤0.10

Table S10. Simple and multiple linear regression models based on the approach of MM-estimation between the FSIQ score and the natural logarithm transformation of OPFRs biomarkers standardized for creatinine categorized by tertiles, in the whole PCB cohort and stratified by child' sex.

	**PCB cohort**	
	**Simple regression (MM-estimation)**	**Multiple regression (MM-estimation)†**
**OPFRs biomarkers:**	**β (95%CI)**	**β (95%CI)**
**DPHP (µg/g crt):**	***N=296***	***N=279***
0.42 ≤ DPHP < 1.09 **VS** DPHP < 0.42	1.30 (-3.27; 5.88)	2.21 (-1.69; 6.12)
DPHP ≥ 1.09 **VS** DPHP < 0.42	-3.34 (-7.86; 1.19)	-2.74 (-6.55; 1.08)
**BDCIPP (µg/g crt):**	***N=296***	***N=279***
-2.25 ≤ BDCIPP < -1.33 **VS** BDCIPP < -2.25	0.06 (-4.52; 4.63)	0.23 (-3.65; 4.11)
BDCIPP ≥ -1.33 **VS** BDCIPP < -2.25	-1.45 (-6.05; 3.16)	-1.22 (-5.13; 2.69)

Abbreviations: FSIQ, Full Scale Intelligence Quotient; β, beta coefficient. †Adjusted for children's body mass index z-score and the highest level of education of the household of the child.
**p-value≤0.05; *p-value≤0.10

Table S11. Simple and multiple linear regression models based on the approach of MM-estimation between the FSIQ score and the natural logarithm transformation of OPFRs biomarkers standardized for creatinine and binary OPFRS biomarkers in the OCC and PCB cohort.

	OCC		PCB cohort	
**Biomarker of exposure**	**Simple regression (MM-estimation)**	**Multiple regression (MM-estimation)†**	**Simple regression (MM-estimation)**	**Multiple regression (MM-estimation)†**
	**β (95%CI); N=264**	**β (95%CI); N=259**	**β (95%CI); N=296**	**β (95%CI); N=279**
**OPFR (μg/g crt):**				
DPHP	-0.88 (-3.18; 1.42)	-0.20 (-2.50; 2.09)	-0.14 (-2.05; 1.77)	-0.21 (-1.83; 1.41)
BDCIPP	-1.42 (-3.09; 0.24)	-1.32 (-2.98; 0.34)	-0.44 (-2.15; 1.26)	-0.55 (-1.99; 0.90)
**BCIPP (μg/g):**				
LOQ≥0.3 vs LOQ<0.3	-	-	0.86 (-3.32; 5.04)	1.84 (-1.67; 5.36)
LOQ≥0.4 vs LOQ<0.4	2.18 (-4.02; 8.37)	1.95 (-4.31; 8.22)	-	-

Abbreviations: FSIQ, Full Scale Intelligence Quotient; β, beta coefficient. †Adjusted for children's sex and body mass index z-score and the highest level of education of the household of the child. **p-value≤0.05; *p-value≤0.10

Table S12. Simple and multiple linear regression models based on the approach of MM-estimation between the FSIQ score and the natural logarithm transformation of OPFRs biomarkers and binary OPFRS biomarkers in the OCC and PCB cohort.

	OCC		PCB cohort	
**Biomarker of exposure**	**Simple regression (MM-estimation)**	**Multiple regression (MM-estimation)†**	**Simple regression (MM-estimation)**	**Multiple regression (MM-estimation)†**
	**β (95%CI); N=264**	**β (95%CI); N=259**	**β (95%CI); N=297**	**β (95%CI); N=280**
**OPFR (μg/g crt):**				
DPHP	-0.68 (-2.80; 1.45)	-0.22 (-2.33; 1.90)	0.05 (-1.92; 2.01)	-0.14 (-1.79; 1.50)
BDCIPP	-1.55 (-3.22; 0.11)*	-1.44 (-3.11; 0.22)*	-0.23 (-2.02; 1.57)	-0.43 (-1.93; 1.07)
**BCIPP (μg/g):**				
LOQ≥0.3 vs LOQ<0.3	-	-	0.92 (-3.35; 5.20)	2.00 (-1.59; 5.60)
LOQ≥0.4 vs LOQ<0.4	2.62 (-3.56; 8.79)	2.28 (-3.97; 8.53)	-	-

Abbreviations: FSIQ, Full Scale Intelligence Quotient; β, beta coefficient. †Adjusted for children's sex and body mass index z-score and the highest level of education of the household of the child. **p-value≤0.05; *p-value≤0.10

Figure S1. Directed acyclic graph (DAG) representing the assumed relationships between the exposure to OPFRs and the outcome neurodevelopment, specific for children.

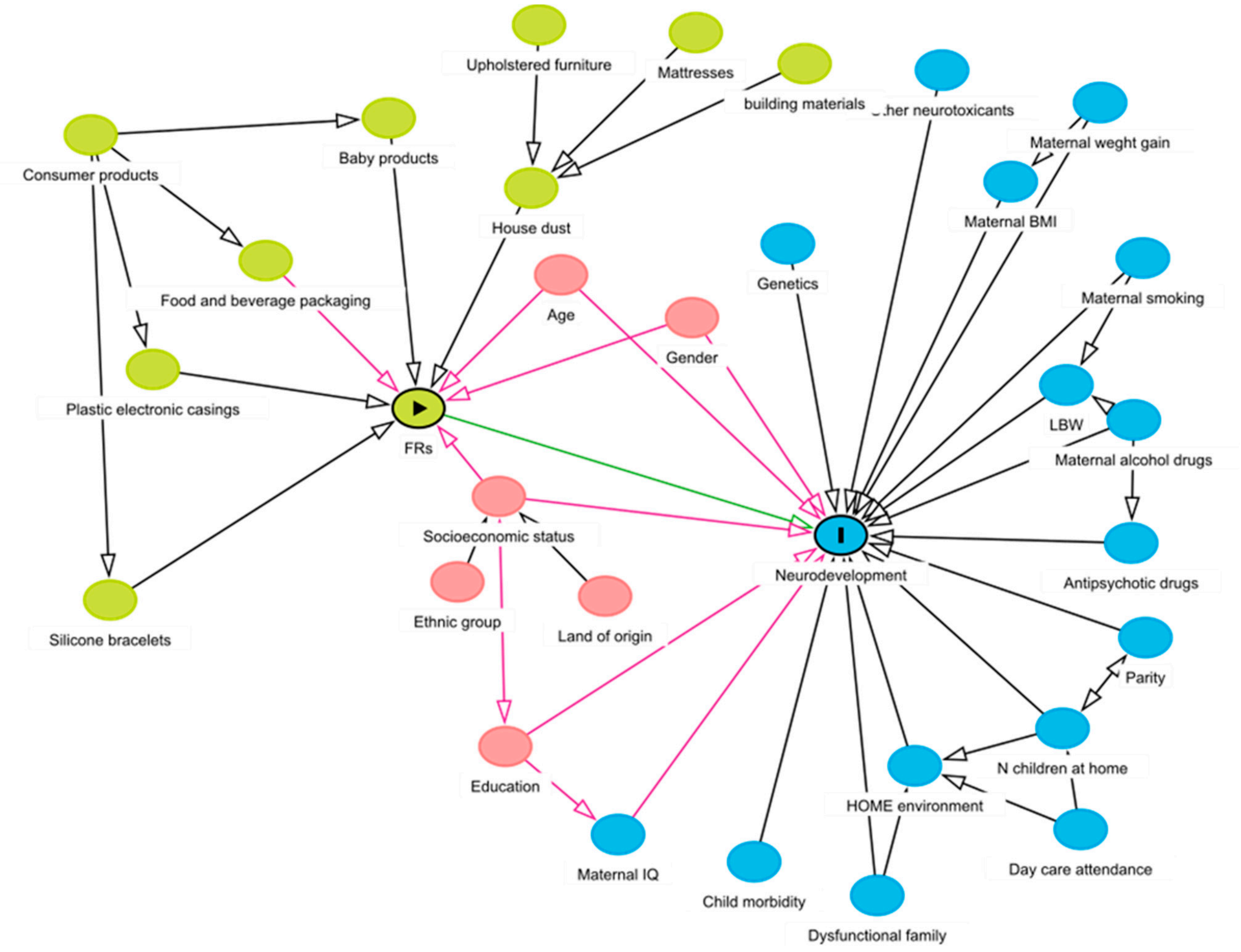

Blue nodes represent the outcome and ancestors of the outcome; green nodes the exposure and ancestors of the exposure; red nodes ancestors of both the exposure and the outcome. The green edge (=arrow) shows the assumed causal path between the exposure and outcome. Abbreviations: FRs, organophosphate flame retardants; LBW, low body weight.
